# U-Shaped Relationship of Rare Earth Element Lanthanum and Oral Cancer Risk: A Propensity Score-Based Study in the Southeast of China

**DOI:** 10.3389/fpubh.2022.905690

**Published:** 2022-05-12

**Authors:** Fa Chen, Qingrong Deng, Yuxuan Wu, Yuying Wu, Jinfa Chen, Yujia Chen, Lisong Lin, Yu Qiu, Lizhen Pan, Xiaoyan Zheng, Lihong Wei, Fengqiong Liu, Baochang He, Jing Wang

**Affiliations:** ^1^Department of Epidemiology and Health Statistics, School of Public Health, Fujian Medical University, Fuzhou, China; ^2^Key Laboratory of Ministry of Education for Gastrointestinal Cancer, Fujian Medical University, Fuzhou, China; ^3^Laboratory Center, School of Public Health, Fujian Medical University, Fuzhou, China; ^4^Department of Oral and Maxillofacial Surgery, The First Affiliated Hospital of Fujian Medical University, Fuzhou, China

**Keywords:** rare earth elements, lanthanum, oral cancer, propensity score analyses, risk assessment

## Abstract

As an important rare earth element (REE) extensively applied to industry, agriculture, and medicine, lanthanum (La) has attracted a host of health concerns. This study aimed to explore the relationship between La exposure and the risk of developing oral cancer through a case-control study with a large sample size. Serum La levels of 430 oral cancer patients and 1,118 healthy controls were detected by inductively coupled plasma mass spectrometry (ICP-MS). The association of La level with the risk of oral cancer was assessed in two ways: (1) as a continuous scale based on restricted cubic splines (RCS); (2) as a priori defined centile categories using multivariate logistic regression model, based on propensity score matching (PSM) and inverse probability of treatment weighting (IPTW). The RCS revealed a non-linear U-shaped relationship between serum La and oral cancer risk. Serum La deficiency or excess was associated with an increased risk of oral cancer. When the La level was analyzed as a categorical variable, a similar U-shaped association was observed. Of note, compared to those with La concentrations of 0.243–0.341 μg/L (reference quantiles, 41st−60th), the risk was increased in those with the lower or higher quantiles (0.132–0.242 μg/L vs. 0.243–0.341 μg/L: OR = 1.80, 95%CI: 1.07–3.02; 0.342–0.497 μg/L vs. 0.243–0.341 μg/L: OR = 2.30, 95%CI: 1.38–3.84). The results were generally consistent with the PSM and IPTW analyses. This preliminary study provides strong evidence that there was a U-shaped relationship between serum La levels and oral cancer risk. Much additional work is warranted to confirm our findings.

## Introduction

Oral cancer, the most common kind of head and neck tumor, is a collective term for malignant tumors that arise in the lip, tongue, gums, floor of the mouth, jaw, buccal mucosa, oral vestibule, and other sides (1). According to the latest data from the International Agency for Research on Cancer (IARC), oral cancers account for 377,713 new cases and 177,757 cancer-related deaths worldwide in 2020 (2). Tobacco smoking, alcohol consumption, and betel nut chewing are well-established as risk factors for oral cancer (3, 4). Nevertheless, these conventional risk factors do not entirely explain the overall risk of oral cancer, particularly among those who lack all these characteristics. It may signal that other exogenous risk factors need to be identified.

Lanthanum (La) is one of the most common elements of REEs (rare earth elements), and it has been widely utilized in diverse fields for decades, including agriculture, catalysis, pharmacology, and electronics (5, 6). Yearly La production in China is 45,000 tons, making it the second-highest element of REEs production in China (7). Studies have revealed that La enters and accumulates in the body *via* numerous routes, including skin absorption, respiratory and dietary intake *via* the oral cavity. A recent study investigated the residual levels of REEs in 14 representative food categories collected from 33 major cities in China and found La being the second most abundant REEs in all food samples (8). Moreover, previous studies revealed that tea, one of the most popular beverages, especially in southern China, contains a certain amount of many rare earth elements including La (9, 10). Long-term ingestion of these foods led to bioaccumulation and continuous exposure to La. Not only that, it has considerable biological activity in biological tissues and organs (11–14). Thus, the public health issue has become increasingly worrisome with the migration and transformation of La in the ecosystem.

Several experimental studies have indicated that La has an anti-cancer effect on several cervical cancer cells, hepatocellular carcinoma cells, and ovarian cancer cells (15–19). Other studies, however, showed that La has carcinogenic potential when its concentration fluctuates in colorectal and hepatic cancer cells (20). Of note, a recent study revealed that soluble La (III) species were found to cause NLRP3 inflammasome activation, which has been proved to be a mechanism for promoting oral cancer (21–23). However, most of those researches were restricted to cell or animal experiments. Hitherto, few studies involved population-based samples (such as blood) to investigate the association between internal La exposure and cancer risk, and even less so, the role of La exposure in oral cancer risk in southeastern China where there is a high production of REEs. Southern China contains large amounts of granite weathering crust, which is enriched in ion-adsorption REEs in lateritic clay deposits and the area accounted for 35% of China's REE production from 1988 to 2009 (24, 25). In this milieu, inhabitants of this region are more likely to experience high and frequent La exposure. Considering the relatively high incidence of oral cancer in this region (26), this study was aimed (1) to detect the serum concentrations of La in a large-sacred sample size with the technology of inductively coupled plasma mass spectrometry (ICP-MS), and (2) to further explore the relationship of La on oral cancer risk using two powerful approaches (propensity score matching (PSM)) and inverse probability of treatment weighting (IPTW) for minimizing the potential confounding effects.

## Materials and Methods

### Study Population

The case-control study was carried out from January 2010 to August 2019 in Fujian Province, China. A total of 430 newly diagnosed cases of oral cancer were enrolled from the First Affiliated Hospital of Fujian Medical University. During the same period, a total of 1,118 healthy controls were recruited from the health examination center of the same hospital.

The following are the inclusion and exclusion criteria of the study: Participants in both case and control groups were (1) aged 20–85 years old; (2) lived in Fujian Province for more than 10 years; and (3) answered questions clearly. All cases were (1) primary oral cancer with post-operative histologically confirmed; (2) had never previously received chemotherapy or radiation therapy. All controls had no history of oncological diseases. If any of the following conditions were met, participants were excluded from the study: (1) with severe systemic infections, severe heart disease, hepatic disease, renal system and hematopoietic system disease, malnutrition of the whole body, or systemic immune diseases; and (2) had taken long-term medications or dietary supplements that would interfere with study outcomes.

This study was conducted in compliance with the Declaration of Helsinki's ethical principles and was authorized by the Fujian Medical University's Institutional Review Board in Fuzhou, China (Approval ID: 2011053).

### Data Collection

Relevant information on each subject was gathered through a face-to-face-based structured questionnaire by trained professional investigators. All the participants signed informed consent. The information we collected mainly included: demographic characteristics (age, gender, occupation, education level, BMI, residence, and family history of cancer), lifestyle habits (smoking, alcohol drinking, and tea consumption), and dietary factors (intake frequency of red meat, fish, seafood, vegetables, and fruits). Smokers were classified as those who smoked more than 100 cigarettes throughout their lives (27). Alcohol drinkers were those who had at least one drink per week for at least 6 months (28). Tea drinkers are defined as the people who drink tea one cup per week at least, lasting for over half a year (28). After the survey, the quality of the questionnaires was reviewed and checked to assure the quality of data and its completeness.

### Blood Sample Collection and La Elemental Detection

Fasting venous blood samples (about 3–5 mL) were donated from the patients on the second day of hospitalization before any drug treatment or examination. Also, the blood samples of the healthy controls were obtained on the day of their physical examination at the same hospital. All samples were centrifuged at 3,000 rpm for 10 min at 4°C and then were preserved at −80°C until measurement.

The detailed methodology for elemental detection can be found in our previous study (29, 30). Briefly, before detection, two preprocessing steps of microwave digestion and acid discharge are necessary. First, 200 μL of serum was accurately measured and combined with 1 mL of nitric acid (HNO_3_) and 4 mL of ultrapure water for microwave digestion (PreeKem, China). The surplus acid was then evaporated at 140°C until the solution was almost dry. The digested samples were diluted with 5% nitric acid to a volume of 10 ml. The concentrations of La were then determined by inductively coupled plasma mass spectrometry (ICP-MS, NexION 350X; Perkin-Elmer, USA).

For quality control, human hair powder (GBW07601a, China) the closest human composition standard for serum La, was utilized as internal standard reference material for continuous monitoring of the efficiency and accuracy of ICP-MS analysis. Briefly, each batch included at least two experimental blanks and two quality control samples. Simultaneously, 12.5% of each batch's samples are chosen at random for parallel sample testing, and the relative standard deviation of the parallel sample testing findings does not exceed 10%.

### Statistical Analysis

Chi-square or Fisher's exact tests were performed to compare baseline characteristics between oral cancer patients and controls. The serum La level was represented as a median (quartile25–quartile75), and the Wilcoxon rank-sum test was used to examine the difference between cases and controls. The association between La levels and oral cancer was assessed using a continuous scale or restricted cubic spline curves (RCS) based on multivariable logistic regression analysis with adjustment for covariates. To balance the best fit and overfitting in the major splines, five knots were chosen at the fifth, 27.5th, 50th, 72.5th, and 95th percentiles of the La distribution based on the lowest Akaike information criterion value. To avoid possible selection bias and balance demographic differences and other confounding variables, propensity score analyses, including propensity score matching (PSM) and inverse probability of treatment weight (IPTW) analyses, were applied. In PSM, the matching ratio was 1:1 ratio, and the caliper was 0.02 with 288 cases successfully matched with 288 healthy controls. Given that PSM can cause sample loss, we also performed the estimated propensity scores to calculate IPTW. After PSM and IPTW, group differences were assessed using standardized mean differences (SMD), with an SMD value of 0.1 deemed balanced.

Furthermore, associations between seven established La classes and oral cancer in unmatched and matched populations were investigated. The categories of La were defined by the 5th, 20th, 40th, 60th, 80th, and 95th centiles with the reference category (41st−60th centiles) determined by the La level linked with the lowest risk of oral cancer in RCS analysis. Finally, multivariate-non-conditional logistic regression (for overall population) and conditional logistic regressions (for PSM and IPTW populations) were utilized to calculate the odds ratios (ORs) and the 95% confidence intervals (CIs) of the relationship between La classes and the risk of oral cancer. A 2-tailed *P*-value < 0.05 was considered statistically significant. All analyses were performed using R software version 4.0.5.

## Results

The study comprised a total of 1,548 participants, with 430 oral cancer patients and 1,118 healthy controls. The baseline characteristics of the study participants are summarized in Supplementary Table 1. Significant differences were observed between the case and control groups in terms of demographic characteristics, lifestyle factors (smoking, alcohol, and tea-drinking), and dietary habits (intake frequency of red meat, fish, seafood, vegetables, and fruits).

**Figure 1:**
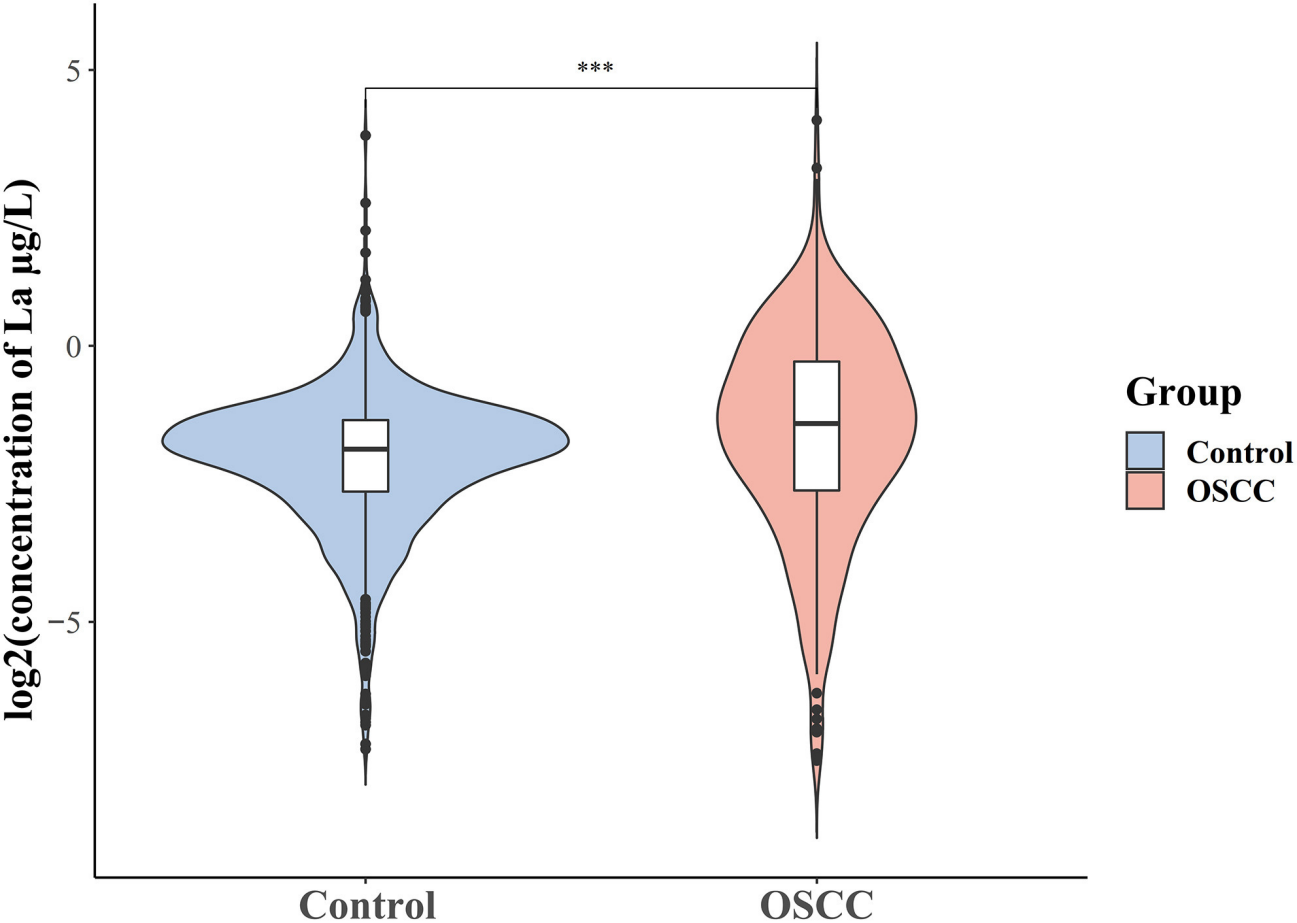
Distribution of serum La in case and control1 groups, for visualization purposes, we log^2^ transformed the measured la concentrations. ^***^P < 0.001 by Wilcoxon rank sum test.

**Figure 2:**
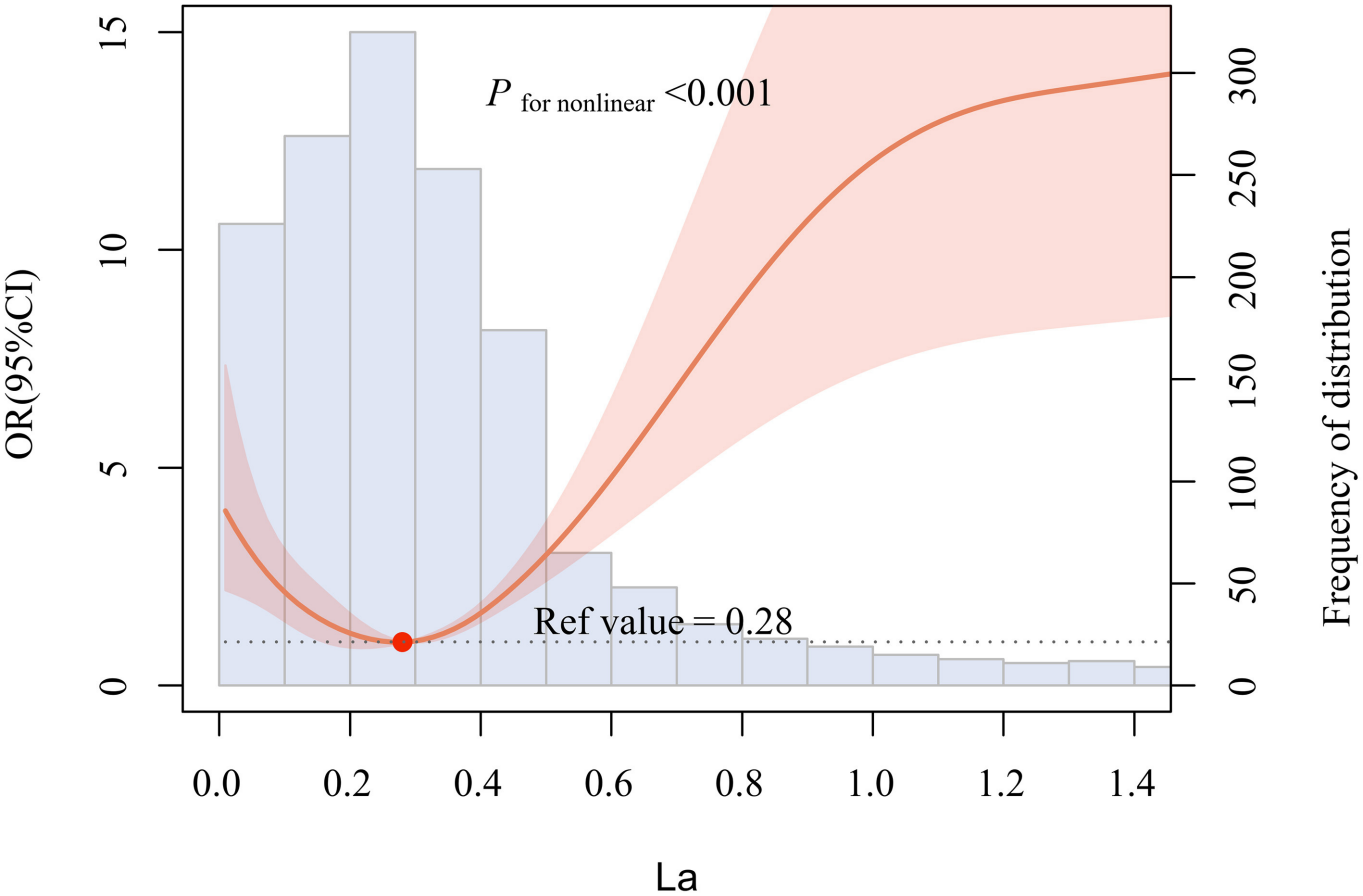
Multivariable-adjusted restricted cubic splines (RCS) according to levels of La on a continuous scale. Solid red lines are multivariable-adjusted odd ratios, with red shading indicating 95% confidence intervals derived from restricted cubic spline regressions with five knots. Reference lines for no association are indicated by the dash lines at an odd ratio of 1.0. The blue regions represent the histogram of the distribution of La. And the solid red dot indicates the concentration of La with the lowest risk of oral cancer. Analyses were adjusted for age, gender, occupation, education level, BMI, residence, family history of cancer, smoking, alcohol and tea drinking, as well as intake frequency of red meat, fish, seafood, vegetables, and fruits.

For purposes of aesthetics and visual interpretation, the concentration of La in the violin plot was log2 transformed. As presented in Figure 1, the level of La in oral cancer patients was significantly higher than that of healthy controls [median (quartile25 to quartile75): −1.4064 (−2.6180 to −0.2816) log2 (μg/L) vs. −1.8722 (−2.6395 to −1.3422) log2 (μg/L), or 0.3772 (0.1629–0.8227) μg/L vs. 0.2732 (0.1605–0.3944) μg/L, *P* < 0.001, data not shown]. Then, the multivariable-adjusted restricted cubic splines revealed a non-linear U-shaped association between serum La and the risk of oral cancer, which indicated that low or high levels of La both increased the risk of oral cancer (*P*_fornon−linear_ < 0.001). The lowest risk was observed at 0.28 μg/L level of La (Figure 2).

**Figure 3:**
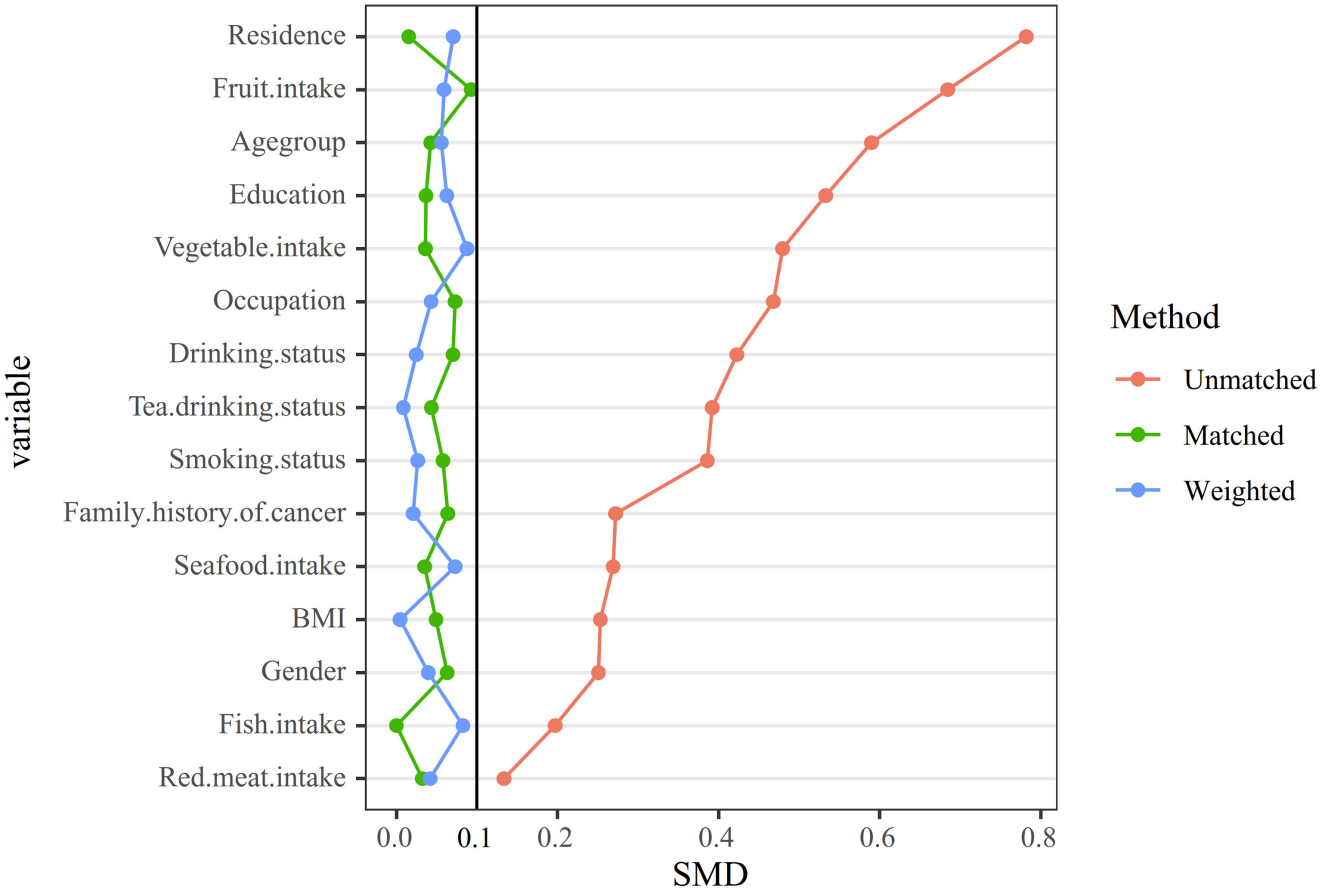
Comparison of the distribution of matching factors between cases and controls before and after matching. Group differences were assessed using standardized mean differences (SMD), with an SMD value of 0.1 considered balanced.

To minimize the potential confounding factors, we performed 1:1 PSM and IPTW analysis to further confirm our observations. After PSM and IPTW, all the covariates were balanced between case and control groups, with all the standardized differences <10% (Table 1; Figure 3). We then defined five equally distributed categories of La by the 20th, 40th, 60th, and 80th centiles. Furthermore, two additional categories were defined by the 5th and 95th centiles to evaluate the highest and lowest levels of La. The La level associated with the lowest risk of oral cancer in restricted cubic spline analysis served as the reference category for these analyses.

**Table 1:** Baseline characteristics of case and control groups in PSM and IPTW population.

**Variables**		**1:1 PSM**			**IPTW**		
		**Control**	**Case**	**SMD**	**Control**	**Case**	**SMD**
*N*		288	288		1,559.4	1,467.6	
Gender	Male	157 (54.5)	166 (57.6)	0.063	831.3 (53.3)	753.3 (51.3)	0.040
	Female	131 (45.5)	122 (42.4)		728.1 (46.7)	714.3 (48.7)	
Agegroup (years)	<60	114 (39.6)	120 (41.7)	0.042	487.3 (31.3)	497.0 (33.9)	0.056
	≥60	174 (60.4)	168 (58.3)		1,072.1 (68.7)	970.6 (66.1)	
Occupation	Farmer	96 (33.3)	106 (36.8)	0.073	707.6 (45.4)	637.6 (43.4)	0.043
	Worker	34 (11.8)	32 (11.1)		175.4 (11.2)	162.6 (11.1)	
	Office worker and others	158 (54.9)	150 (52.1)		676.4 (43.4)	667.4 (45.5)	
Education level	Illiteracy	30 (10.4)	29 (10.1)	0.037	269.5 (17.3)	241.8 (16.5)	0.062
	Primary-middle school	184 (63.9)	189 (65.6)		990.6 (63.5)	907.5 (61.8)	
	High school and above	74 (25.7)	70 (24.3)		299.3 (19.2)	318.3 (21.7)	
BMI	18.5–23.9	161 (55.9)	168 (58.3)	0.049	834.0 (53.5)	781.5 (53.2)	0.005
	<18.5 or ≥24	127 (44.1)	120 (41.7)		725.5 (46.5)	686.2 (46.8)	
Residence	Rural	204 (70.8)	202 (70.1)	0.015	1,214.0 (77.9)	1,098.6 (74.9)	0.071
	Urban	84 (29.2)	86 (29.9)		345.4 (22.1)	369.0 (25.1)	
Family history of cancer	No	250 (86.8)	256 (88.9)	0.064	1,406.6 (90.2)	1,314.6 (89.6)	0.021
	Yes	38 (13.2)	32 (11.1)		152.8 (9.8)	153.0 (10.4)	
Smoking status	No	187 (64.9)	179 (62.2)	0.058	1,050.5 (67.4)	1,006.7 (68.6)	0.026
	Yes	101 (35.1)	109 (37.8)		508.9 (32.6)	460.9 (31.4)	
Drinking status	No	214 (74.3)	205 (71.2)	0.07	1,207.4 (77.4)	1,121.0 (76.4)	0.025
	Yes	74 (25.7)	83 (28.8)		352.0 (22.6)	346.6 (23.6)	
Tea drinking status	No	187 (64.9)	181 (62.8)	0.043	1,071.0 (68.7)	1,013.8 (69.1)	0.009
	Yes	101 (35.1)	107 (37.2)		488.4 (31.3)	453.8 (30.9)	
Red meat intake	≥3 times	73 (25.3)	69 (24.0)	0.032	429.6 (27.5)	377.2 (25.7)	0.042
(per week)	<3 times	215 (74.7)	219 (76.0)		1,129.8 (72.5)	1,090.4 (74.3)	
Seafood intake	≥1 times	162 (56.2)	167 (58.0)	0.035	792.7 (50.8)	692.6 (47.2)	0.073
(per week)	<1 times	126 (43.8)	121 (42.0)		766.7 (49.2)	775.0 (52.8)	
Fish intake	≥3 times	159 (55.2)	159 (55.2)	<0.001	747.8 (48.0)	643.4 (43.8)	0.083
(per week)	<3 times	129 (44.8)	129 (44.8)		811.6 (52.0)	824.2 (56.2)	
Vegetable intake	≥2 times	109 (37.8)	104 (36.1)	0.036	418.4 (26.8)	452.1 (30.8)	0.088
(per day)	<2 times	179 (62.2)	184 (63.9)		1,141.0 (73.2)	1,015.5 (69.2)	
Fruit intake	≥3 times	183 (63.5)	170 (59.0)	0.093	690.9 (44.3)	693.4 (47.2)	0.059
(per week)	<3 times	105 (36.5)	118 (41.0)		868.5 (55.7)	774.2 (52.8)	

**Figure 4:**
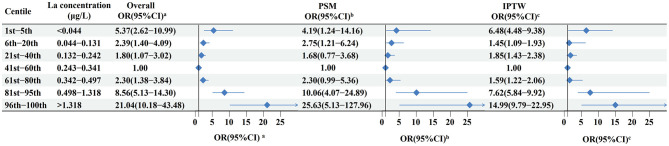
Odd ratios for oral cancer according to categories of levels of La in the total study population, 1:1 PSM population, and IPTW population. **(a)** Calculated from multivariable logistic regression models, with adjustment for age, gender, occupation, education level, BMI, residence, family history of cancer, smoking, alcohol and tea drinking, as well as intake frequency of red meat, fish, seafood, vegetables, and fruits. **(b,c)** Calculated from conditional logistic regression models.

Of note, a U-shaped dose-response relationship was still observed when La was transformed into a categorical variable (Figure 4). Among the overall study population, compared with individuals with the concentration of La of 0.243–0.341 μg/L (41st−60th centiles), the multivariable-adjusted OR was 1.80 (95%CI 1.07–3.02) for individuals with lower quantiles of La (21st−40th centiles: 0.132–0.242 μg/L) and 2.30 (95%CI 1.38–3.84) for those with higher quantiles of La (61st−80th centiles: 0.243–0.341 μg/L). Similar results were observed when using PSM and IPTW analysis, with a markedly elevated risk at the higher and lower levels (Figure 4).

## Discussion

To the best of our knowledge, this is the first large-scale retrospective, case-control study to explore the relationship between serum La and oral cancer risk using propensity score-based analysis. Our study suggested that both too low and too high concentrations of La, either as a continuous or as a categorical variable, were associated with an increased risk of oral cancer. These findings provide strong evidence to support a U-shaped non-linear relationship between serum La and oral cancer risk, rather than a simple linear one.

Although there is a relative paucity of research on the role of La in cancer risk and also no direct explanation for our findings, a recent study indicated that the biological effect of La on hepatocellular carcinoma cells HepG2 and colorectal carcinoma cells HT-29 varied with its concentration, with low concentrations (<70 μM) promoting cell proliferation and high concentrations (180 μM) inhibiting cell growth. The same pattern of effect was also observed in the HT-29 cells (20). These findings therefore indirectly support our results of a U-shaped association between La and oral cancer risk. Interestingly, the majority of *in vitro* cell experiments indicated that La or its derivatives have anticancer properties. Yu et al. (15), found that La ions could suppress cell proliferation and trigger apoptosis in cervical cancer cells by regulating let-7a, miR-34a, and their downstream genes. La citrate complex could inhibit proliferation and promote apoptosis in hepatocellular carcinoma SMMC-7721 cells and cervical cancer Hela cells (16, 17). Several possible explanations may account for this, including causing cell arrest at G0/G1 phase, inhibiting DNA of tumor cell synthesis, and inducing apoptosis through the mitochondrial-mediated apoptotic pathway (31–33). Nevertheless, it was also worth mentioning that excessively high concentrations of La are neurotoxic (34–36). The possible mechanisms mainly include the following: disruption of intracellular calcium homeostasis (37), formation of reactive oxygen species (35) as well as the disruption of physiological functions like the glutamate-glutamine cycle (38) or various signaling pathways such as the PI3K/Akt and NF-κB signaling pathway (12, 36). Soluble La (III) species have the ability to activate NLRP3 inflammasome, and the latter was regarded as a key mediator of inflammation in promoting the development of oral cancer (21–23). These findings provide new insight into the action mechanism of La on cancer. The precise mechanism of the observed linking between high La level and oral cancer risk is difficult to determine at the current stage, which merits further studies.

A synergistic effect of LaCl_3_ on cisplatin-induced inhibition of ovarian cancer cell proliferation and tumor growth has been found (18, 19). The application of La_2_O_3_ nanoparticles can also improve the therapeutic benefits of radiation therapy and chemotherapy with temozolomide in the treatment of glioblastoma (39). All these observations suggested that La complexes might be effective candidate medications in the prevention and treatment of malignant tumors (40, 41). However, the dosage of La complexes appears to be the critical component in switching the biological and anticancer effects on and off (20). In this population-based epidemiological study, we observed that serum La concentration exhibits a non-linear U-shaped association with the risk of OSCC, and the lowest risk was at the concentration of 0.243–0.341 μg/L. Our results provide an intriguing clue that an appropriate range of La concentration (0.243–0.341 μg/L) might be a good emerging approach for the prevention and treatment of oral cancer.

The strength of this study is to utilize two powerful approaches (PSM and IPTW analyses) to significantly reduce potential selection bias and confounding effects and to provide the first epidemiological evidence of a non-linear association between serum La and the risk of oral cancer in the Asian population. However, certain limitations should be mentioned. Firstly, due to the case-control design of this study, the causal direction of the associations cannot be settled, due to the lack of a temporal relationship. Thus, our findings need to be further validated in multi-center, longitudinal population cohorts, whilst *in vitro* and *in vivo* research on underlying biological mechanisms is also warranted. Secondly, the current study only used a single measurement of serum La levels instead of repeated measurements, which may not completely reflect La bioaccumulation or long-term La exposure, and replication in diverse samples (such as hair, nails, etc.) and identifying specific forms of La are therefore needed. Third, although our study provides a reference range of serum La concentrations for the lowest risk of oral cancer, identifying the pharmacological effects is challenging at this stage, and much more extensive research will need to be performed.

In summary, the present study suggests, for the first time, a non-linear U-shaped association between serum La levels and oral cancer risk, with both excess and deficient serum La exhibiting unfavorable effects. These observations shed new light on the role of serum La in oral cancer and have important public health implications as environmental La exposure is a growing concern. In parallel, further research is needed to uncover potential mechanisms of La, which will facilitate the use of La in the diagnosis and treatment of oral cancer and provide new techniques to enhance tumor prevention.

## Data Availability Statement

The raw data supporting the conclusions of this article will be made available by the authors, without undue reservation.

## Ethics Statement

This study was conducted in compliance with the Declaration of Helsinki's Ethical Principles and was authorized by the Fujian Medical University's Institutional Review Board in Fuzhou, China (Approval ID: 2011053). The patients/participants provided their written informed consent to participate in this study.

## Author Contributions

JW, BH, and FL participated in the design of the study. YuyW, YuxW, YC, LL, and YQ were responsible for recruitment and interviewing participants. LP, XZ, and LW contributed to sample collection. JW, JC, and FC performed laboratory experiments. QD and YuxW analyzed the data. FC, JW, and QD wrote the manuscript and which was revised by all authors. All authors contributed to the article and approved the submitted version.

## Funding

This study was funded by the High-Level Talents Research Start-Up Project of Fujian Medical University (No. XRCZX2018001) and Central Government-Led Local Science and Technology Development Special Project (No. 2020L3009).

## Conflict of Interest

The authors declare that the research was conducted in the absence of any commercial or financial relationships that could be construed as a potential conflict of interest.

## Publisher's Note

All claims expressed in this article are solely those of the authors and do not necessarily represent those of their affiliated organizations, or those of the publisher, the editors and the reviewers. Any product that may be evaluated in this article, or claim that may be made by its manufacturer, is not guaranteed or endorsed by the publisher.

## References

[1] Peres M Macpherson L Weyant R Daly B Venturelli R Mathur M. Oral diseases: a global public health challenge. Lancet. (2019) 394:249–60. 10.1016/S0140-6736(19)31146-8 31327369

[2] International Agency for Research on Cancer. Cancer Today. Available online at: https://gco.iarc.fr/today/home (accessed March 31, 2022).

[3] Chen F Lin L Yan L Liu F Qiu Y Wang J. Nomograms and risk scores for predicting the risk of oral cancer in different sexes: a large-scale case-control study. J Cancer. (2018) 9:2543–8. 10.7150/jca.24431 30026853 PMC6036893

[4] Guha N Warnakulasuriya S Vlaanderen J Straif K. Betel quid chewing and the risk of oral and oropharyngeal cancers: a meta-analysis with implications for cancer control. Int J Cancer. (2014) 135:1433–43. 10.1002/ijc.28643 24302487

[5] González V Vignati D Pons M Montarges-Pelletier E Bojic C Giamberini L. Lanthanide ecotoxicity: first attempt to measure environmental risk for aquatic organisms. Environ Pollut (Barking, Essex: 1987). (2015) 199:139–47. 10.1016/j.envpol.2015.01.020 25645063

[6] Gwenzi W Mangori L Danha C Chaukura N Dunjana N Sanganyado E. Sources, behaviour, and environmental and human health risks of high-technology rare earth elements as emerging contaminants. Sci Total Environ. (2018) 636:299–313. 10.1016/j.scitotenv.2018.04.235 29709849

[7] Han G Tan Z Jing H Ning J Li Z Gao S. Comet assay evaluation of lanthanum nitrate DNA damage in C57-ras transgenic mice. Biol Trace Elem Res. (2021) 199:3728–36. 10.1007/s12011-020-02500-5 33403576

[8] Dai Y Sun S Li Y Yang J Zhang C Cao R. Residual levels and health risk assessment of rare earth elements in Chinese resident diet: a market-based investigation. Sci Total Environ. (2022) 828:154119. 10.1016/j.scitotenv.2022.154119 35227721

[9] Luo H Wang W Wang T Hong H Zhou N. Composition and distribution patterns of rare earth elements in Fujian teas. Wei Sheng Yan Jiu. (2014) 43:953–958. 10.19813/j.cnki.weishengyanjiu.2014.06.013 25603605

[10] Wang H Chen X Ye J Jia X Zhang Q He H. Analysis of the absorption and accumulation characteristics of rare earth elements in Chinese tea. J Sci Food Agric. (2020) 100:3360–3369. 10.1002/jsfa.10369 32134117

[11] Lacour B Lucas A Auchère D Ruellan N de Serre Patey N Drüeke T. Chronic renal failure is associated with increased tissue deposition of lanthanum after 28-day oral administration. Kidney Int. (2005) 67:1062–9. 10.1111/j.1523-1755.2005.00171.x 15698446

[12] Yan L Yang J Yu M Lu Y Huang L Wang J. Lanthanum chloride induces neuron damage by activating the nuclear factor-kappa B signaling pathway in activated microglia. Metallomics. (2019) 11:1277–87. 10.1039/c9mt00108e 31187842

[13] Xiao X Yong L Jiao B Yang H Liang C Jia X. Postweaning exposure to lanthanum alters neurological behavior during early adulthood in rats. Neurotoxicology. (2021) 83:40–50. 10.1016/j.neuro.2020.12.012 33359004

[14] Nikolov I Joki N Vicca S Patey N Auchère D Benchitrit J. Tissue accumulation of lanthanum as compared to aluminum in rats with chronic renal failure–possible harmful effects after long-term exposure. Nephron Exp Nephrol. (2010) 115:e112–21. 10.1159/000313492 20424489

[15] Yu L Xiong J Guo L Miao L Liu S Guo F. The effects of lanthanum chloride on proliferation and apoptosis of cervical cancer cells: involvement of let-7a and miR-34a microRNAs. Biometals. (2015) 28:879–90. 10.1007/s10534-015-9872-6 26209160

[16] Chen B Hu Z Chen B Li B. Effects and mechanism of Lanthanum Citrate on the proliferation and apoptosis of hepatocellular carcinoma cell line SMMC-7721. Turk J Gastroenterol. (2020) 31:264–71. 10.5152/tjg.2020.18800 32343239 PMC7197929

[17] Su X Zheng X Ni J. Lanthanum citrate induces anoikis of Hela cells. Cancer Lett. (2009) 285:200–9. 10.1016/j.canlet.2009.05.018 19679391

[18] Wang F Zhu Y Fang S Li S Liu S. Effect of lanthanum chloride on tumor growth and apoptosis in human ovarian cancer cells and xenograft animal models. Exp Ther Med. (2018) 16:1143–8. 10.3892/etm.2018.6299 30116365 PMC6090291

[19] Fang S Zhang P Chen X Liu F Wang F. Lanthanum chloride sensitizes cisplatin resistance of ovarian cancer cells PI3K/Akt pathway. Front Med. (2021) 8:776876. 10.3389/fmed.2021.776876 34977076 PMC8714849

[20] Benedetto A Bocca C Brizio P Cannito S Abete M Squadrone S. Effects of the rare elements lanthanum and cerium on the growth of colorectal and hepatic cancer cell lines. Toxicol In Vitro. (2018) 46:9–18. 10.1016/j.tiv.2017.09.024 28954213

[21] Zheng R Wang L Wu X Song P Wang Y Zhang H. Biotransformation of soluble-insoluble lanthanum species and its induced NLRP3 inflammasome activation and chronic fibrosis. Environ Pollut (Barking, Essex: 1987). (2021) 284:117438. eng. 10.1016/j.envpol.2021.117438 34058500

[22] Scuderi SA Casili G Basilotta R Lanza M Filippone A Raciti G. NLRP3 inflammasome inhibitor BAY-117082 reduces oral squamous cell carcinoma progression. Int J Mol Sci. (2021) 22:108. 10.3390/ijms222011108 34681768 PMC8540383

[23] Yao Y Shen X Zhou M Tang B. Periodontal pathogens promote oral squamous cell carcinoma by regulating ATR and NLRP3 inflammasome. Front Oncol. (2021) 11:722797. eng. 10.3389/fonc.2021.722797 34660289 PMC8514820

[24] Chen H Chen Z Chen Z Ma Q Zhang Q. Rare earth elements in paddy fields from eroded granite hilly land in a southern China watershed. PLoS ONE. (2019) 14:e0222330. 10.1371/journal.pone.0222330 31509591 PMC6738641

[25] Xu C Kynický J Smith MP Kopriva A Brtnický M Urubek T. Origin of heavy rare earth mineralization in South China. Nat Commun. (2017) 8:14598. 10.1038/ncomms14598 28220784 PMC5321793

[26] Lei L Zheng R Peng K Si L Peng J Cai W. Incidence and mortality of oral and oropharyngeal cancer in China 2015. Chin J Cancer Res. (2020) 32:1–9. 10.21147/j.issn.1000-9604.2020.01.01 32194299 PMC7072017

[27] Hashibe M Brennan P Benhamou S Castellsague X Chen C Curado MP. Alcohol drinking in never users of tobacco, cigarette smoking in never drinkers, and the risk of head and neck cancer: pooled analysis in the International Head and Neck Cancer Epidemiology Consortium. J Natl Cancer Inst. (2007) 99:777–89. 10.1093/jnci/djk179 17505073

[28] Chen F He BC Yan LJ Liu FP Huang JF Hu ZJ. Tea consumption and its interactions with tobacco smoking and alcohol drinking on oral cancer in southeast China. Eur J Clin Nutr. (2017) 71:481–5. eng. 10.1038/ejcn.2016.208 28176772

[29] He B Wang J Lin J Chen J Zhuang Z Hong Y. Association between rare earth element cerium and the risk of oral cancer: a case-control study in Southeast China. Front Public Health. (2021) 9:647120. 10.3389/fpubh.2021.647120 34113597 PMC8186664

[30] Chen F Wang J Chen J Yan L Hu Z Wu J. Serum copper and zinc levels and the risk of oral cancer: a new insight based on large-scale case-control study. Oral Dis. (2019) 25:80–6. 10.1111/odi.12957 30107072

[31] Asadi Z Nasrollahi N Karbalaei-Heidari H Eigner V Dusek M Mobaraki N. Investigation of the complex structure, comparative DNA-binding and DNA cleavage of two water-soluble mono-nuclear lanthanum(III) complexes and cytotoxic activity of chitosan-coated magnetic nanoparticles as drug delivery for the complexes. Spectrochim Acta A Mol Biomol Spectr. (2017) 178:125–35. 10.1016/j.saa.2017.01.037 28178588

[32] Neelima Poonia K Siddiqui S Arshad M Kumar D. *In vitro* anticancer activities of Schiff base and its lanthanum complex. Spectrochim Acta A Mol Biomol Spectr. (2016) 155:146–54. 10.1016/j.saa.2015.10.015 26619196

[33] Heffeter P Jakupec M Körner W Chiba P Pirker C Dornetshuber R. Multidrug-resistant cancer cells are preferential targets of the new antineoplastic lanthanum compound KP772 (FFC24). Biochem Pharmacol. (2007) 73:1873–86. 10.1016/j.bcp.2007.03.002 17445775 PMC3371634

[34] Wu J Yang J Lu X Jin C Wu S Zhang L. Lanthanum chloride impairs the blood-brain barrier integrity by reduction of junctional proteins and upregulation of MMP-9 in rats. Biol Trace Elem Res. (2019) 187:482–91. 10.1007/s12011-018-1402-2 29876795

[35] Yuan L Qu Y Li Q An T Chen Z Chen Y. Protective effect of astaxanthin against LaO nanoparticles induced neurotoxicity by activating PI3K/AKT/Nrf-2 signaling in mice. Food Chem Toxicol. (2020) 144:111582. 10.1016/j.fct.2020.111582 32673631

[36] Zheng L Zhang J Yu S Ding Z Song H Wang Y. Lanthanum chloride causes neurotoxicity in rats by upregulating miR-124 expression and targeting PIK3CA to regulate the PI3K/Akt signaling pathway. Biomed Res Int. (2020) 2020:5205142. 10.1155/2020/5205142 32461997 PMC7222569

[37] Yu M Yang J Gao X Sun W Liu S Han Y. Lanthanum chloride impairs spatial learning and memory by inducing [Ca] overload, mitochondrial fission-fusion disorder and excessive mitophagy in hippocampal nerve cells of rats. Metallomics Integr Biometal Sci. (2020) 12:592–606. eng. 10.1039/c9mt00291j 32163055

[38] Du Y Yang J Yan B Bai Y Zhang L Zheng L. Lanthanum enhances glutamate-nitric oxide-3',5'-cyclic guanosine monophosphate pathway in the hippocampus of rats. Toxicol Ind Health. (2016) 32:1791–1800. eng. 10.1177/0748233715590517 26071434

[39] Lu V Jue T McDonald K. Cytotoxic lanthanum oxide nanoparticles sensitize glioblastoma cells to radiation therapy and temozolomide: an *in vitro* rationale for translational studies. Sci Rep. (2020) 10:18156. 10.1038/s41598-020-75372-3 33097778 PMC7584621

[40] Kapoor S. Lanthanum and its rapidly emerging role as an anti-carcinogenic agent. J Cell Biochem. (2009) 106:193. 10.1002/jcb.21985 19097134

[41] Zhang J Li Y Hao X Zhang Q Yang K Li L. Recent progress in therapeutic and diagnostic applications of lanthanides. Mini Rev Med Chem. (2011) 11:678–94. 10.2174/138955711796268804 21679137

