# Supplementary material for: U-Shaped Relationship of Rare Earth Element Lanthanum and Oral Cancer Risk: A Propensity Score-Based Study in the Southeast of China
Source: Front Public Health. 2022 May 12;10:905690. doi: 10.3389/fpubh.2022.905690 (PMC9133527; doi:10.3389/fpubh.2022.905690)
Supplement: Supplementary file 1 [file Table_1.DOCX]

Supplementary Material

Supplementary Table 1

Supplementary Table 1 Baseline characteristics of case and control groups in overall population

| Variables | | Overall (%) | Control (%) | Case (%) | *P* value | χ^2^ |
| --- | --- | --- | --- | --- | --- | --- |
| N |  | 1548 | 1118 | 430 |  |  |
| Gender | Male | 812 (52.5) | 548 (49.0) | 264 (61.4) | <0.001 | 19.083 |
|  | Female | 736 (47.5) | 570 (51.0) | 166 (38.6) |  |  |
| Agegroup (years) | ＜60 | 460 (29.7) | 248 (22.2) | 212 (49.3) | <0.001 | 109.363 |
|  | ≥60 | 1088 (70.3) | 870 (77.8) | 218 (50.7) |  |  |
| Occupation | Farmer | 696 (45.0) | 572 (51.2) | 124 (28.8) | <0.001 | 62.579 |
|  | Worker | 173 (11.2) | 110 (9.8) | 63 (14.7) |  |  |
|  | Office worker and others | 679 (43.9) | 436 (39.0) | 243 (56.5) |  |  |
| Education level | Illiteracy | 271 (17.5) | 241 (21.6) | 30 (7.0) | <0.001 | 80.757 |
|  | Primary-middle school | 994 (64.2) | 722 (64.6) | 272 (63.3) |  |  |
|  | High school and above | 283 (18.3) | 155 (13.9) | 128 (29.8) |  |  |
| BMI | 18.5-23.9 | 855 (55.2) | 579 (51.8) | 276 (64.2) | <0.001 | 19.303 |
|  | <18.5 or ≥24 | 693 (44.8) | 539 (48.2) | 154 (35.8) |  |  |
| Residence | Rural | 1229 (79.4) | 989 (88.5) | 240 (55.8) | <0.001 | 202.321 |
|  | Urban | 319 (20.6) | 129 (11.5) | 190 (44.2) |  |  |
| Family history of cancer | No | 1408 (91.0) | 1043 (93.3) | 365 (84.9) | <0.001 | 26.688 |
|  | Yes | 140 (9.0) | 75 (6.7) | 65 (15.1) |  |  |
| Smoking status | No | 1063 (68.7) | 824 (73.7) | 239 (55.6) | <0.001 | 47.403 |
|  | Yes | 485 (31.3) | 294 (26.3) | 191 (44.4) |  |  |
| Drinking status | No | 1195 (77.2) | 920 (82.3) | 275 (64.0) | <0.001 | 59.315 |
|  | Yes | 353 (22.8) | 198 (17.7) | 155 (36.0) |  |  |
| Tea drinking status | No | 1102 (71.2) | 852 (76.2) | 250 (58.1) | <0.001 | 49.429 |
|  | Yes | 446 (28.8) | 266 (23.8) | 180 (41.9) |  |  |
| Red meat intake | ≥ 3 times | 452 (29.2) | 345 (30.9) | 107 (24.9) | 0.021 | 5.363 |
| (per week) | < 3 times | 1096 (70.8) | 773 (69.1) | 323 (75.1) |  |  |
| Seafood intake | ≥ 1 times | 802 (51.8) | 538 (48.1) | 264 (61.4) | <0.001 | 21.916 |
| (per week) | < 1 times | 746 (48.2) | 580 (51.9) | 166 (38.6) |  |  |
| Fish intake | ≥ 3 times | 758 (49.0) | 517 (46.2) | 241 (56.0) | 0.001 | 11.943 |
| (per week) | < 3 times | 790 (51.0) | 601 (53.8) | 189 (44.0) |  |  |
| Vegetable intake | ≥ 2 times | 413 (26.7) | 231 (20.7) | 182 (42.3) | <0.001 | 74.508 |
| (per day) | < 2 times | 1135 (73.3) | 887 (79.3) | 248 (57.7) |  |  |
| Fruit intake | ≥ 3 times | 693 (44.8) | 400 (35.8) | 293 (68.1) | <0.001 | 131.533 |
| (per week) | < 3 times | 855 (55.2) | 718 (64.2) | 137 (31.9) |  |  |
